# Effect of the distal histidine on the peroxidatic activity of monomeric cytoglobin

**DOI:** 10.12688/f1000research.5971.1

**Published:** 2015-04-07

**Authors:** Penny Beckerson, Dimitri Svistunenko, Brandon Reeder

**Affiliations:** 1 School of Biological Sciences, University of Essex, Colchester, Essex, CO4 3SQ, UK

**Keywords:** Cytoglobin, distal histidine, peroxide, ferryl, hexacoordinate, peroxidase, monomer

## Abstract

The reaction of hydrogen peroxide with ferric human cytoglobin and a number of distal histidine variants were studied. The peroxidase activity of the monomeric wildtype protein with an internal disulfide bond, likely to be the form of the protein *in vivo*, exhibits a high peroxidase-like activity above that of other globins such as myoglobin. Furthermore, the peroxidatic activity of wildtype cytoglobin shows increased resistance to radical-based degradation compared to myoglobin. The ferryl form of wildtype cytoglobin is unstable, but is able to readily oxidize substrates such as guaiacol. In contrast distal histidine mutants of cytoglobin (H81Y and H81V) show very low peroxidase activity but enhanced radical-induced degradation. Therefore, the weakly bound distal histidine appears to modulate ferryl stability and limit haem degradation. These data are consistent with a role of a peroxidase activity of cytoglobin in cell stress response mechanisms.

## Introduction

Cytoglobin (Cygb) is a haem protein ubiquitously expressed in vertebrate cells ^1, 2^. Cygb has a coordinating distal histidine in the deoxygenated ferrous form, giving a hexacoordinate haem iron similar to neuroglobin (Ngb) ^1, 3, 4^. Several possible physiological functions of Cygb have been proposed including nitric oxide (NO) dioxygenase activity, involvement in collagen synthesis and oxidative stress response ^5– 8^. The mechanisms by which Cygb affects cellular responses to oxidative stress are unknown, however it has been suggested that protection of cells may occur by Cygb functioning as a scavenger of reactive oxidative species (ROS) or as a peroxidase ^9, 10^. *In vivo* studies have shown that Cygb expression is up-regulated by hydrogen peroxide suggesting a role in oxidative stress protection ^6^. This is supported by Cygb knockdown studies showing sensitisation of cells to oxidative stress, with over-expression providing protection ^5^. The specific role of Cygb in these stress responses is unclear.

The reaction of Cygb with peroxides has not been extensively studied. However, the reactions of pentacoordinate globins, such as myoglobin (Mb) and haemoglobin (Hb), with hydrogen peroxide have been well characterised ^11, 12^. The reaction of ferrous or ferric Mb and Hb with peroxides leads to the formation of the ferryl species ([Fe ^4+^=O ^2-^] ^2+^). Protein based radicals are also formed in the reaction of ferric Mb or Hb with peroxides. Unlike classical peroxidase enzymes, the radical in these systems is inherently unstable leading to oxidative modifications to the haem and protein. In pentacoordinate globins the distal histidine (e.g. H64 in Mb) is important in providing a hydrogen bond to the partially deprotonated bound peroxide (Fe ^3+^-OOH ^-^) ^13^. This facilitates the scission of the di-oxygen bond to form the ferryl iron and porphyrin cation radical (Compound I), followed by a rapid migration and partial decay of the radical to form compound II ^14– 16^. Proteins that lack the distal residue (e.g. H64Q in Mb) lead to a transient stabilisation of the peroxide-bound intermediate (Compound 0) over the millisecond timescale ^13, 17^. In Ngb the hexacoordinate configuration of the haem iron prevents the formation of a ferryl species ^18^.

This study examines the mechanism of the interaction of Cygb with peroxide and the role of the distal histidine in such reactions. Our data show that the monomeric wildtype (WT) Cygb, when reacting with peroxides, is characterised by a high peroxidase activity (in comparison to other globins) and that the ferryl formed (compound II) is more unstable than seen in Mb or Hb. The distal histidine is particularly important for this peroxidase activity since the mutants lacking this residue show a low substrate oxidation and high rates of haem degradation. This study supports a role of Cygb peroxidase activity in stress responses *in vivo*.

## Materials and methods

### Cytoglobin engineering, expression and purification

The human Cygb gene was purchased from OriGene (via Cambridge Bioscience, UK), transferred from the cloning vector into expression vector pET28a (Merck Millipore, UK), expressed and purified as previously described ^19, 20^. Cygb pET28a plasmid was mutated using site-directed mutagenesis based on a modified Agilent Quikchange II method to give the H81V and H81Y mutation. Initial denaturation was 95°C for 2 min followed by 20 cycles of 95°C for 50 s, 55°C for 60 s and 68°C for 13 min. Primers were purchased from Eurofins, UK. The mutated Cygb were also expressed and purified using the same protocol as for the wildtype (WT) protein ^19^. The monomeric, dimeric and polymeric Cygb were separated using G75 Superdex column (GE Healthcare) equilibrated with 0.1 M sodium phosphate buffer (Fischer, UK) pH 7.4. Fractions were collected corresponding to absorbance peaks at 280 nm. Monomeric Cygb variants were used for all subsequent experiments.

### UV-Vis spectroscopy

A Varian Cary 5E UV-vis spectrophotometer was used to measure UV-visible spectra. Extinction coefficients of the Cygb distal histidine mutants were calculated as previously described using HPLC ^20^.

Far UV CD spectra of monomeric WT, H81V and H81Y Cygb (5 μM) in 20 mM sodium phosphate buffer (pH 7.4) were measured using an Applied Photophysics Chirascan CD spectrophotometer between wavelengths 180–300 nm in a 1 ml quartz cuvette at 20°C. Scan speed was 60 nm min ^-1^. Three spectra were taken and averaged and corrected for buffer baseline.

### Peroxidase activity assay

Monomeric WT, H81V and H81Y Cygb (5 μM) in 0.1 M sodium phosphate buffer pH 7.4 were reacted with hydrogen peroxide (5 mM) in the presence of 8.94 mM guaiacol (0.1% v/v, Sigma, UK). The peroxidase activity of Cygb was monitored by the formation of tetraguaiacol (470 nm) due to the oxidation of guaiacol by the ferryl iron ( equation 1):

4 Guaiacol + 4 H2O2→Globin Tetraguaiacol + 8 H2O.    (1)

The reaction was followed using an Agilent 8453 diode array spectrophotometer.

### Reactions of cytoglobin with hydrogen peroxide

Ferric monomeric WT, H81V and H81Y Cygb (50 μM), in 20 mM phosphate buffer (pH 7.4), were reacted with a range of hydrogen peroxide (Sigma, UK) concentrations (0–5 mM) and incubated at room temperature for 18 hours. The samples were analysed by both non-reducing (4% stacking, 12% resolving) and reducing SDS-PAGE (4% stacking and 12% resolving) gel electrophoresis.

Oxidative damage to the haem in these samples was measured by reverse phase HPLC using an Agilent HP1100 HPLC fitted with a diode array spectrophotometer. The column used was a Zorbax StableBond 300SB C3 250 mM × 4.6 mM fitted with a 12 mm × 4.6 mm guard column. Solvent A was 0.1% trifluoroacetic acid (TFA) (Fisher, UK) and solvent B was acetonitrile (VWR, UK) containing 0.1% TFA. The gradient started at 35% solvent B for 10 min, increasing to 37% for 5 min, increasing to 40% for 1 minute with a final increment to 43% for 10 min. The flow rate was 1 ml min ^-1^ and the temperature was 25°C ^21^. The haem concentration was calculated from the area under the peak at ~14 min.

The kinetics of the formation of ferryl Cygb was measured using an Applied Photophysics SX20 stopped-flow spectrometer fitted with a diode array unit. Ferric monomeric WT, H81V and H81Y Cygb (5 µM after mixing, in 50 mM sodium phosphate buffer pH 7.4) were rapidly mixed with a range of hydrogen peroxide concentrations (0–10 mM final). Absorbance changes following mixing were recorded using the proSX software and kinetics fitted globally using the Applied Photophysics ProKinetist software.

### Electron paramagnetic resonance spectroscopy

Monomeric WT, H81V and H81Y Cygb (80 μM) in 20 mM sodium phosphate buffer (pH 7.4) were reacted with hydrogen peroxide, either at 1:1 at (80 μM) or 1:10 (800 μM) protein:hydrogen peroxide ratio in Wilmad SQ EPR tubes (Wilmad Glass). The reaction was halted by flash-freezing in dry ice cooled methanol at various time points (0, 20, 45, 90, 180 s). The samples were stored in liquid nitrogen (77 K) prior to measurement. Electron paramagnetic resonance (EPR) spectra were taken using a Bruker EMX EPR spectrophotometer (X-band) equipped with a spherical high-quality resonator SP9703 and an Oxford Instruments liquid helium system. The modulation frequency was 100 kHz. Accurate *g* values were obtained using the built-in microwave frequency counter and a 2,2-diphenyl-1-picrylhydrazyl powder standard, the *g* value for which is g = 2.0027 ± 0.0002 ^22, 23^.

## Results

### Optical characteristics of cytoglobin distal mutation

**Figure 1.**
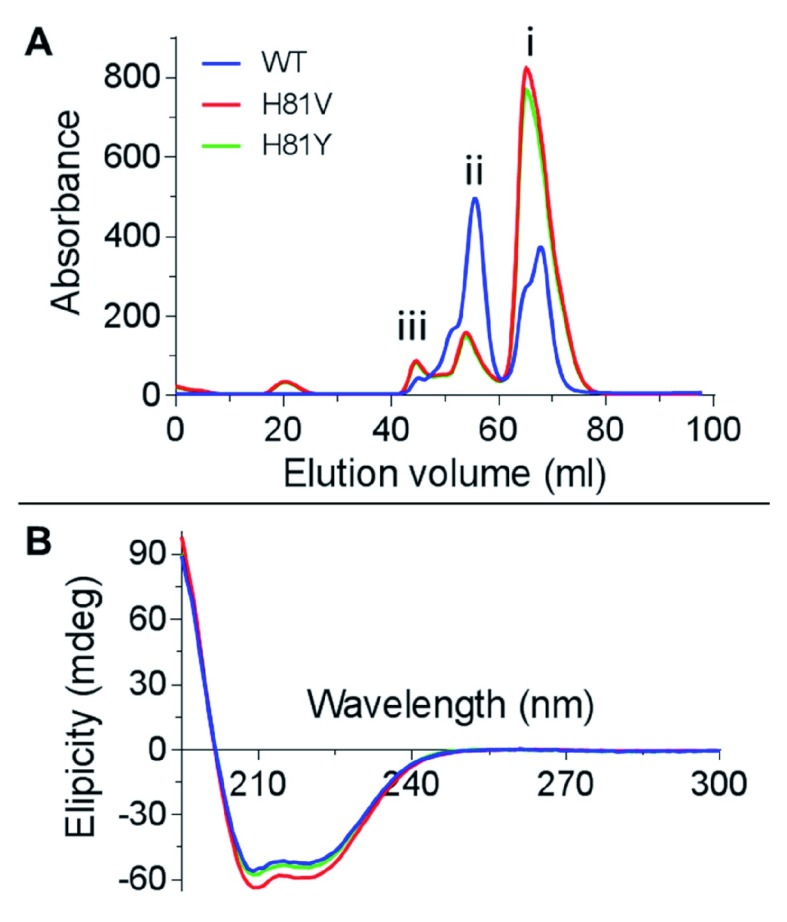
Cytoglobin mutant conformation and secondary structure. ( **A**) Size exclusion chromatography (G-75 Superdex) of recombinant WT cytoglobin showing two major peaks, (i) at 56 ml corresponding to the 43 kDa dimer and (ii) at 67 ml corresponding to the 21 kDa monomer respectively. A small peak at ~45 ml corresponding to a minor fraction of polymerised Cygb. The mutant Cygb show the same peaks, however the peak corresponding the monomer (i) is the most prominent. ( **B**) CD spectra of Cygb distal histidine mutants displaying characteristic double minima at 209 and 222 nm identifying α-helical content.

The expression of WT Cygb displays mixed monomer and dimer conformations as previously described ^20^. However, the expressed H81V and H81Y mutants were predominantly monomeric as shown in the protein elution profiles from gel filtration ( Figure 1A). Herein only monomeric protein was used in this study. The circular dichroism (CD) spectra of Cygb distal histidine mutants are shown in Figure 1B. All proteins display the typical double minima at 209 and 222 nm characteristic of protein structures that is predominantly α-helical, essentially identical to the WT protein as previously reported ^24^. Therefore the mutations did not significantly alter the secondary structure and the recombinant protein has folded correctly.

**Figure 2.**
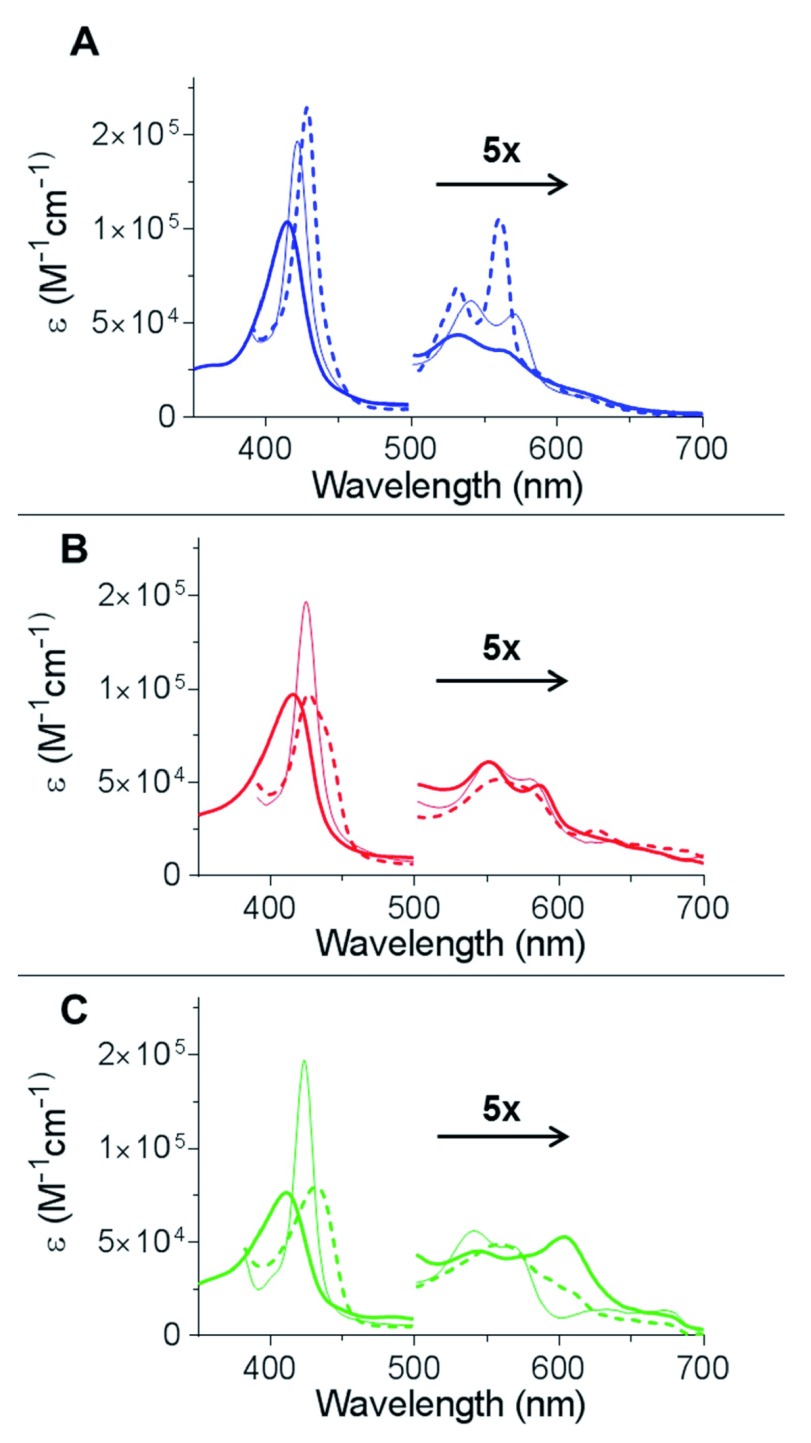
UV-Visible spectra of the WT cytoglobin and two distal histidine mutants. ( **A**) The optical spectra of WT monomeric Cygb as previously described ^20^. ( **B**) The optical spectra of H81V Cygb. The ferric (solid) has a Soret of 415 nm and low intensity α and β peaks at 550 and 588 nm respectively. The reduction of the protein by dithionite (dashed) led to a bathochromic shift with a broadening of the Soret accompanied by appearance of a single broad αβ peak. The binding of CO (thin solid) led to a hypsochromic shift of the Soret with an increase in intensity. This is accompanied with the appearance of the α and β peaks to 580 and 550 nm respectively. ( **C**) The optical spectra of H81Y Cygb. The ferric (solid) has a Soret of 411 nm and three peaks in the visible region at 500, 543 and 605 nm. The reduction of the protein by dithionite (dashed) led to a bathochromic shift of the Soret accompanied by an appearance of a single αβ band with a shoulder. The binding of CO (thin solid) led to a hypsochromic shift of the Soret with an increase in intensity. This is accompanied with the appearance of the α and β peaks at 570 and 542 nm respectively.

The optical spectra of Cygb are shown in Figure 2A in the ferric, deoxyferrous and ferrous-CO bound forms. These spectra are typical of those previously shown for the WT protein ^19, 24^. The ferrous-CO spectrum of H81V and H81Y Cygb is essentially identical to that of the WT protein with a Soret of 423 nm and α and β peaks of 570 and 542 nm respectively ^19, 24^. Mutation of the distal histidine results in significant changes of the optical properties of the protein when in the absence of exogenous ligands ( Figure 2B and Figure 2C respectively). In the deoxyferrous state both of the distal histidine mutants exhibit only a single αβ band with a low intensity shoulder at higher wavelength in comparison to the WT which has two distinct peaks. The α and β bands of the WT protein is characteristic of a hexacoordinated haem iron, similar to that observed in Ngb or cytochrome c ^3, 25^. The two mutant proteins deoxyferrous optical characteristics are more typical of a pentacoordinate haem iron ligation as seen in Mb and Hb ^11^. However, the broad Soret band of deoxyferrous H81V Cygb is wider than observed with the H81Y protein and Mb or Hb, suggesting that H81V has a subpopulation of hexacoordinated species. In the ferric oxidation state the H81Y mutant displays three peaks in the visible region ( Figure 2C) and exhibits a green appearance due to the 600 nm band compared to the orange-brown appearance of the other ferric proteins ^11^. Other haem proteins with a distal tyrosine also display these characteristic absorbance peaks such as the homodimeric haemoglobin from *Mycobacterium tuberculosis* (HbN) ^26^ or in myoglobin with a distal tyrosine mutation ^27^.

### EPR spectroscopy

**Figure 3.**
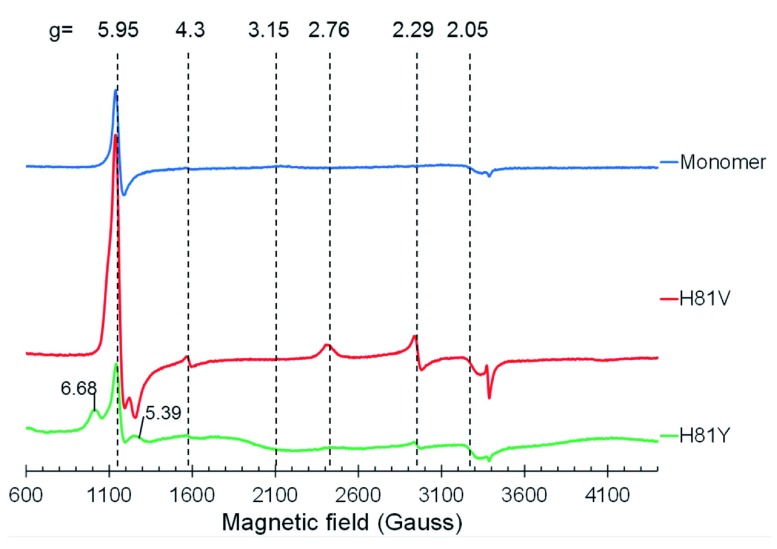
EPR spectra of cytoglobin distal histidine mutants. EPR spectra of ferric cytoglobin (80 µM): wild type monomer with internal disulfide bond (upper spectrum), histidine 81(E7) to valine mutation (middle spectrum) and histidine 81 to tyrosine mutation (lower spectrum). The wild type protein is predominantly in a HisF8–Fe(III)–HisE7 co-ordination (g _3_ = 3.20, g _2_ = 2.05, g _1_ is off scale) as previously reported ^19^. The H81V protein is mainly high spin with a HisF8–Fe(III)–H _2_O configuration with a minor (but sharper) LS component (g _3_ = 2.76, g _2_ = 2.29, g _1_ is off scale). The H81Y protein is again predominantly HS ferric haem rhombic signal with g = 6.68 and g = 5.39.

The EPR spectrum of monomeric ferric WT Cygb is shown in Figure 3 and is consistent with that previously reported ^20^, comprising of a high spin (HS) signal at g = 5.95, a low-spin (LS) signal at g = 3.15 and a small component at g = 4.3 representative of iron ferric ion in rhombic coordination mainly originating from damaged haem ^28^. Both of the distal histidine mutants exhibit different EPR spectra to the WT monomer. WT Cygb is partially HS showing a weak distal histidine coordination in the ferric form of the protein as previously described ^19, 20^. In the H81V mutant the HS signal is much greater compared to the WT protein, indicating the H81V is primarily pentacoordinate in the ferric state. This is also the case for the H81Y variant. Although the HS signal intensity for the H81Y appears lower, there is a notable contribution from the rhombic HS ferric haem signal with g _x_ = 6.68 and g _y_ = 5.39 which makes the whole EPR signal wider than the axial EPR line at g _┴_ = 5.95. This accounts for a higher spin concentration at a similar intensity of the EPR signal.

Unlike WT Cygb, the mutants do not exhibit the broad LS ferric haem form spectrum with g = 3.15, which accounts for a significant fraction of protein in the WT. There is a different LS ferric haem EPR signal seen in both mutants, with g = 2.76 and g = 2.29. The concentration of this haem form is 5.2 times higher in the H81V mutant as compared to the H81Y mutant. The g-factors of this LS ferric haem form are close to some bis-His LS forms observed in other proteins ^29, 30^. We speculate that a different residue can be the cause of this LS ferric haem state, likely resulting from a nitrogen ligand that can be positioned at the 6th haem coordination site. The best candidate for such a residue is the nearby arginine (R84). One can speculate why a LS ferric haem EPR signal is stronger in the valine mutant compared with the tyrosine mutant – most likely because tyrosine is larger allowing lower R84 occupancy.

### Peroxidase activity and reaction kinetics

**Figure 4.**
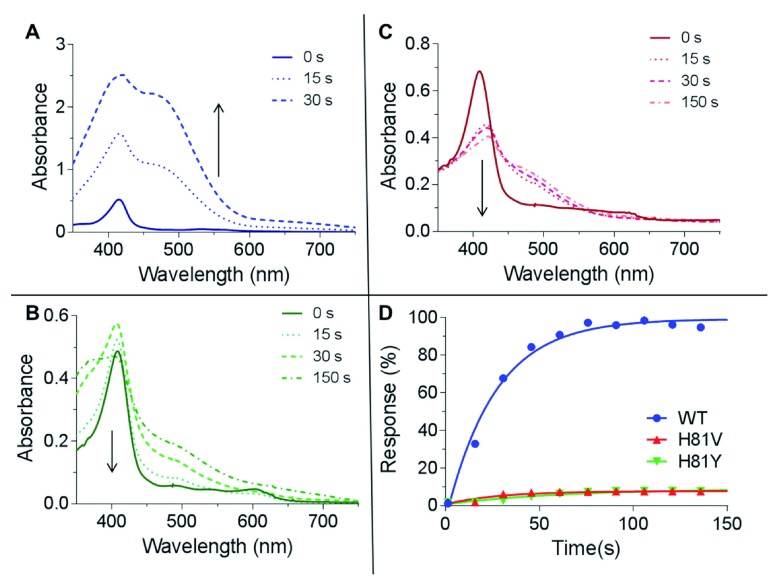
Peroxidase activity of cytoglobin distal histidine mutants using guaiacol oxidation assay. ( **A**) Spectra of the oxidation of guaiacol (8.9 mM) from the reaction of WT Cygb (5 µM) and H _2_O _2_ (5 mM), showing the rapid formation of tetrameric guaiacol. ( **B** and **C**) Conditions are for those described in ( **A**) except with H81V ( **B**) and H81Y ( **C**) variants of Cygb. There is little guaiacol oxidation observed but extensive haem bleaching within the time course of the experiment. ( **D**) Time course of guaiacol oxidation showing a high peroxidase activity of WT Cygb.

The reaction of WT Cygb with hydrogen peroxide does not form a ferryl species that can be monitored by standard optical spectroscopy, unlike other haem proteins such as Mb and Hb ^17, 31^. Therefore the peroxidatic activity was monitored using the guaiacol peroxidase activity assay which uses guaiacol as a substrate for ferryl Cygb, generating a tetrameric oxidation product of guaiacol that can be monitored at 470 nm ( Figure 4A) ^32^. The formation of the tetrameric oxidation product of guaiacol following the reaction of H81V and H81Y with hydrogen peroxide is negligible in comparison to the WT protein ( figure 4D). This initially suggests that there is limited ferryl formation at the peroxide concentration studied (5 mM). However, the bleaching of the haem moiety, which is particularly rapid with the H81V protein, signifies that the guaiacol is not reacting with the ferryl haem iron hence radical damage from redox cycling is prominent. Therefore an alternative method was used to attempt to measure the formation of ferryl haem iron directly using stopped-flow spectroscopy.

**Figure 5.**
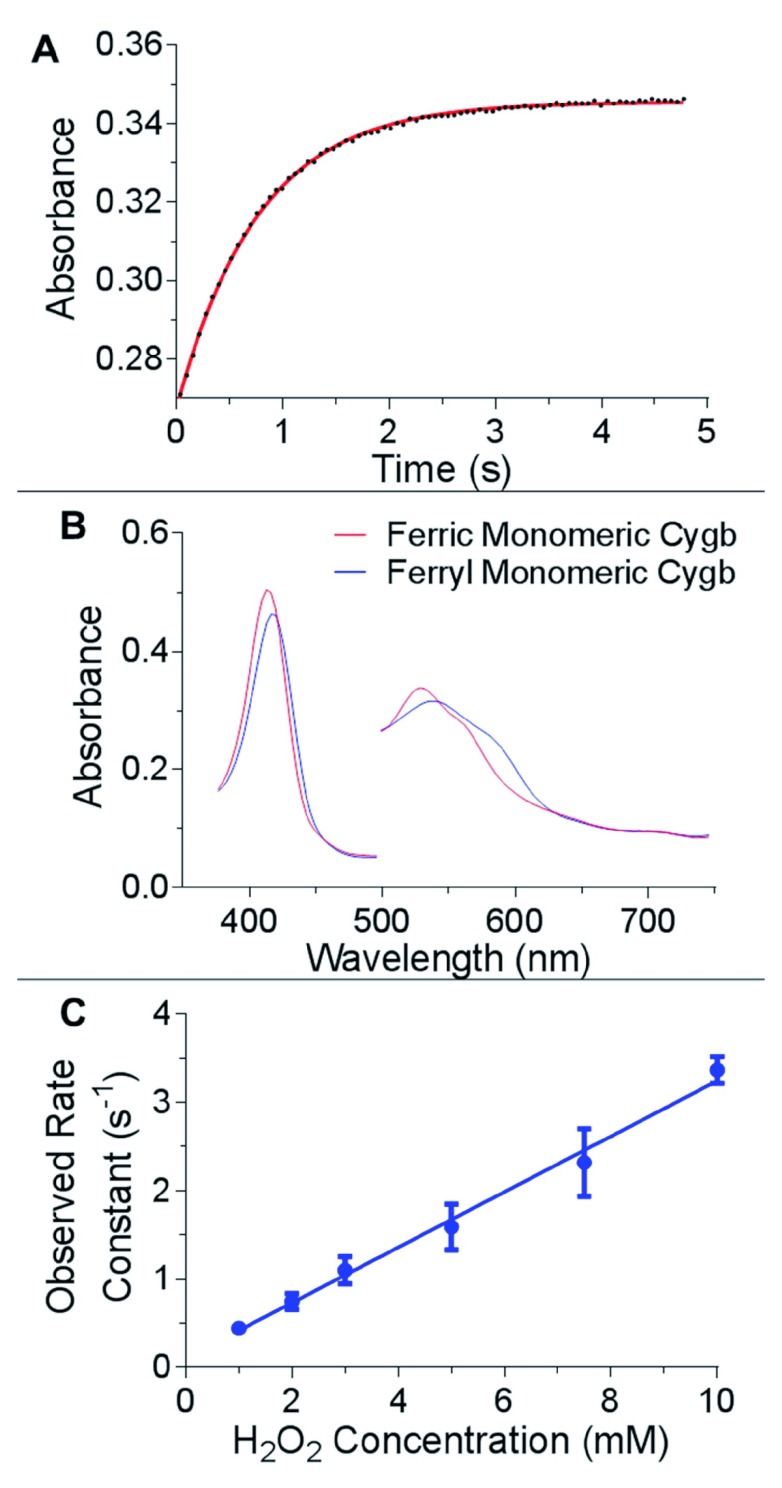
Ferryl formation of cytoglobin distal histidine mutants monitored by rapid reaction kinetics. ( **A**) Time course of monomeric WT Cygb (5 µM) reacting with H _2_O _2_ (5 mM). The changes in absorbance at 420 nm over time were fitted to a single exponential. ( **B**) UV-visible changes observed during the reaction of monomeric Cygb (5 µM) with 5 mM H _2_O _2_, identifying the ferryl species of Cygb with a Soret peak of 418 nm and α and β peaks of 542 and 562 nm respectively. ( **C**) The hydrogen peroxide concentration dependence on ferryl formation observed rate constants of monomeric WT Cygb (5 µM) giving a second order rate constant of 312.3 ± 16.3 M ^-1^s ^-1^.

The reaction of monomeric Cygb with high concentrations of hydrogen peroxide (1 to 10 mM) displayed monophasic time course ( Figure 5A). The spectrum of the ferryl species has a Soret peak of 418 nm and α and β peaks of 562 and 542 nm respectively ( Figure 5B), consistent with the spectrum of ferryl Mb ^31^. The rate of formation of the ferryl species was hydrogen peroxide concentration dependent with a second order rate constant of 312.3 ± 16.3 M ^-1^s ^-1^ ( Figure 5C). This value is very similar to previously reported values of the second order rate constant of myoglobin reacting with hydrogen peroxide (170 M ^-1^s ^-1^ ^33^ and 267 M ^-1^s ^-1^ ^16^). There were no changes in amplitude in response to hydrogen peroxide concentration.

**Figure 6.**
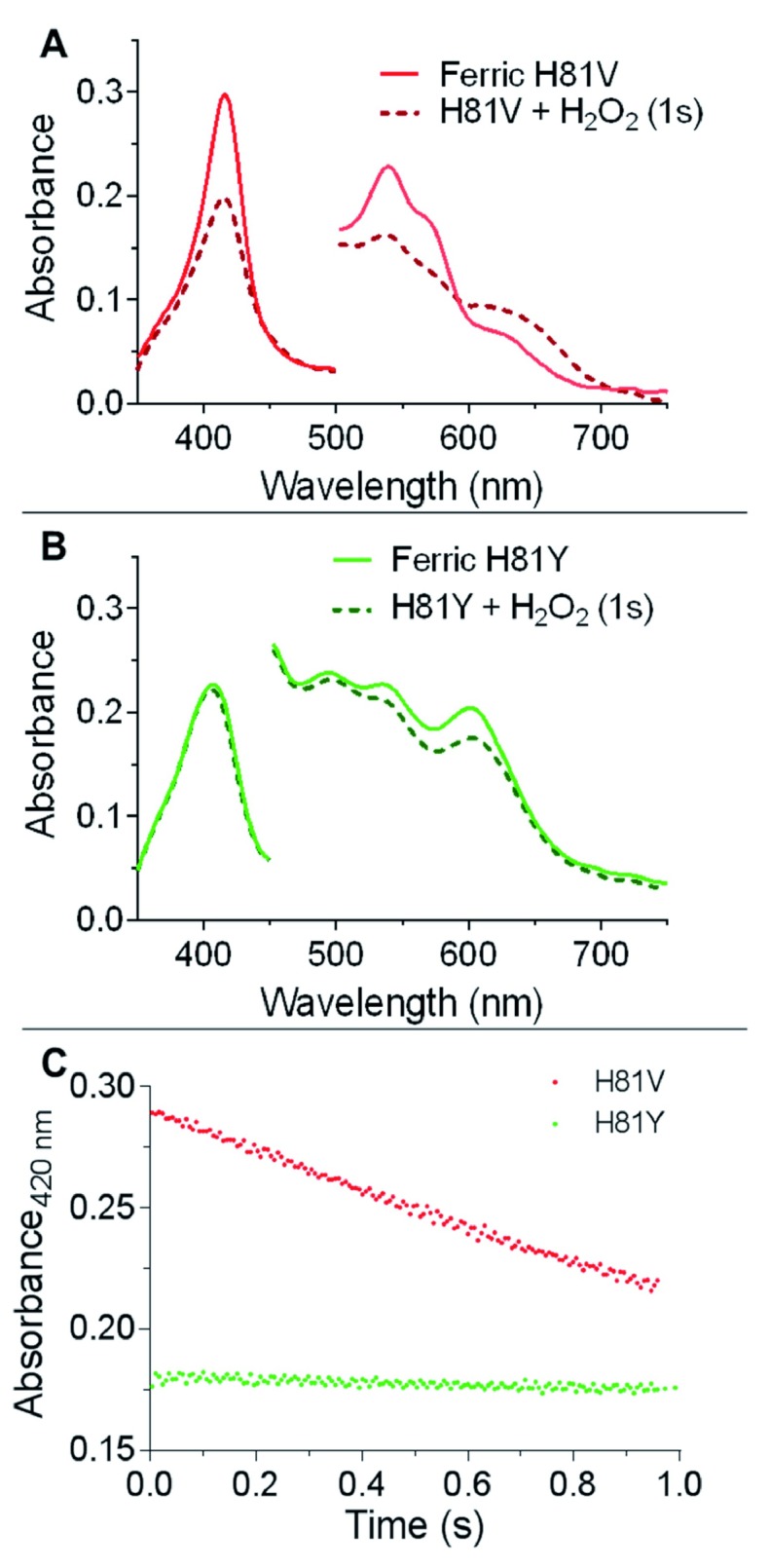
Cytoglobin distal histidine mutants reaction with hydrogen peroxide. ( **A**) UV-visible changes of H81V (5 µM) due to the reaction with 100 mM H _2_O _2_ over the time course of 1 s. ( **B**) UV-visible changes of H81Y (5 µM) due to the reaction with 100 mM H _2_O _2_ over the time course of 1 s. ( **C**) Time course of H81V and H81Y (5 µM) reacting with H _2_O _2_ (40 mM). The changes in absorbance at 420 nm over time were fitted linearly.

The reaction of the distal histidine mutants with hydrogen peroxide showed no measurable formation of ferryl haem iron, with the main optical changes indicative of haem degradation from radical damage ( Figure 6A–C). There is a transient increase in absorbance at ~600–700 nm for H81V, which may be suggestive of an oxidative modified intermediate species.

### Peroxide-induced damage to cytoglobin protein and haem

**Figure 7.**
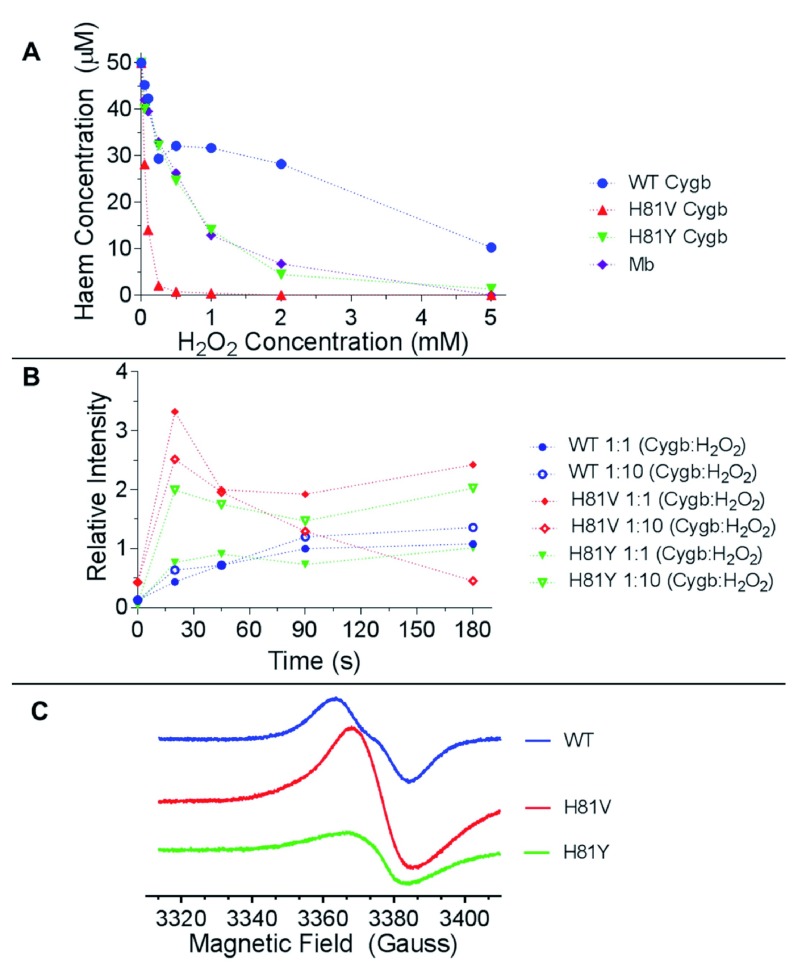
Effect of distal histidine mutation on peroxide-induced haem degradation and radical formation. ( **A**) HPLC analysis of cytoglobin (50 µM) following reaction with H _2_O _2_ (0–5 mM) showing unmodified haem concentration. ( **B**) Time course of radical concentration formed in the reaction of cytoglobin (80 µM) and H _2_O _2_ (at either 1:1 or 1:10 molar excess), as measured by the EPR spectroscopy. ( **C**) Example EPR spectra (t=90 s) exhibiting a primarily doublet radical line shape of WT cytoglobin compared to the singlet line shape of H81Y and H81V.

To monitor radical damage to the haem of Cygb, the protein (50 µM) was reacted with hydrogen peroxide (0–5 mM) at 25°C and the oxidative damage to the protein assessed after reaction was complete by HPLC analysis and gel electrophoresis. The HPLC results ( Figure 7) show that WT Cygb is more resistant to haem damage compared to Mb, with 2500 µM peroxide required to degrade or modify 50% of the haem compared to 500 µM for Mb. However, the distal histidine mutants were more susceptible to peroxide induced haem damage than the WT protein with H81Y showing 50% haem damage at 500 µM peroxide and 60 µM for H81V (~1:1 peroxide: protein ratio). Interestingly Mb and H81Y show identical resistance to peroxide induced haem damage at all peroxide concentrations studied.

**Figure 8.**
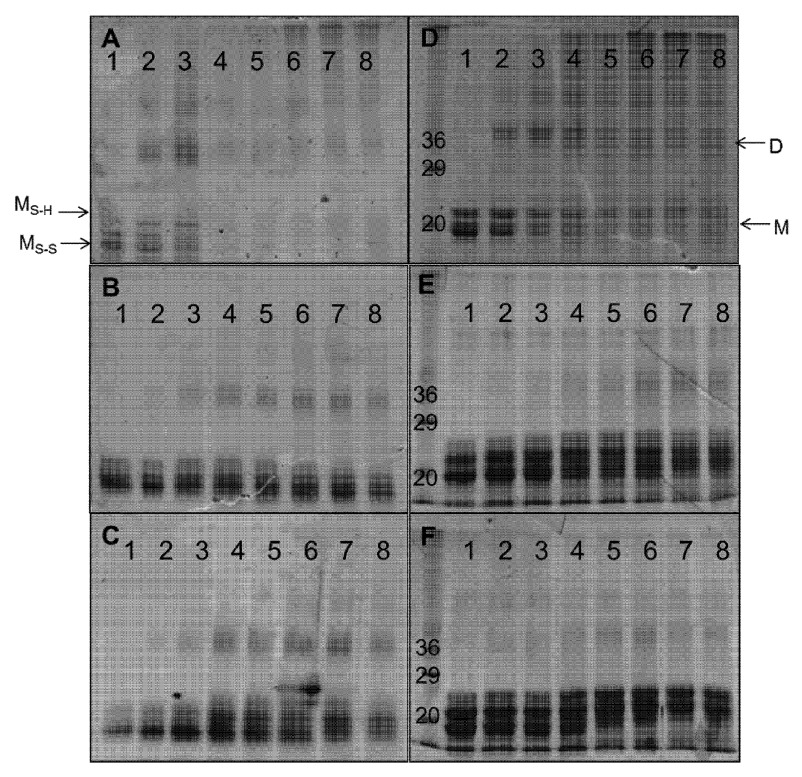
Gel electrophoresis of product of cytoglobin distal histidine variants following the reaction with hydrogen peroxide. Cygb (50 μM) was reacted for 18 hours with 0, 50, 100, 250, 500, 1000, 2500, 5000 μM H _2_O _2_ (lanes 1–8 respectively). SDS-PAGE analysis of WT, H81V and H81Y Cygb are shown in **A**, **B** and **C** respectively and non-reducing PAGE analysis of WT, H81V and H81Y Cygb are shown in **D**, **E** and **F** respectively. The monomer and dimer conformations are shown as (M) and (D) respectively.

The non-reducing PAGE analysis of monomeric Cygb before hydrogen peroxide treatment showed two bands that correspond to the monomeric protein, one with an intramolecular disulfide bond and the other with a reduced disulfide bond ( Figure 8A) as described previously ^20^. These bands lose intensity upon reaction with low concentrations of hydrogen peroxide (50–100 μM) accompanied with the appearance of bands representative of dimeric and higher order conformers. At hydrogen peroxide concentrations above 1 mM the protein forms aggregates as shown by minimal gel migration.

Both of the distal histidine mutants exhibit bands similar to that of the WT monomeric protein showing the presence and absence of an intramolecular disulfide bond ( Figure 8B and Figure 8C). The mutants appear to be more resistant to peroxide induced protein damage than the WT with significant concentrations of the monomeric forms at even 5 mM peroxide (100× excess). There is dimerization of the mutant proteins at hydrogen peroxide ratios as low as 1:1 (protein:peroxide) similar to the WT protein. These dimers are still apparent at high peroxide concentrations in the mutant protein unlike the WT Cygb.

Monitoring the same reaction products using reducing gels (SDS-PAGE, Figure 8D–F) showed a well-defined band at ~21 kDa indicative of monomeric Cygb. In comparison with the non-denaturing gels the monomer and dimer bands lose intensity with increasing peroxide concentrations, which is more evident in the WT protein relative to the mutants. As the denaturing gel eliminates disulfide bonds the peroxide-induced formation of dimers and higher oligomers are likely to arise from covalent di-tyrosine links resulting from the termination of surface-exposed tyrosine radicals on two or more protein chains as have been reported for Mb and Hb ^34, 35^.

### Peroxide-induced radical formation and stability

EPR spectra of the radicals formed from the reaction of Cygb with H _2_O _2_ are shown in Figure 7C. All proteins studied react with hydrogen peroxide to form protein-based radicals. However, the shape of the radical signal in WT differs significantly from the two mutants. While the WT radical exhibits a doublet-like EPR signal, the two mutants show a singlet EPR signal. An EPR signal that lacks a hyperfine structure is always difficult to interpret. However, with respect to the 15–18 G wide singlet EPR spectrum of the mutants, it should be noted that similar EPR signals of protein-bound free radicals have been reported in a number of occasions and were recently interpreted as superposition of several non-specific protein radicals formed as a result of disperse radical propagation during the radical character decay process ^36^. The WT monomer Cygb, on the other hand, shows a doublet-like EPR line shape pointing to a possibility that the radical originates more likely from a single site on the protein, rather than many different sites. The formation rate and stability of the protein radical for WT and H81Y proteins are essentially identical for both 1:1 and 1:10 protein:peroxide ratios and are stable over the period of 180 seconds examined. The formation rate of the radical in the H81V protein is marginally larger but the radical itself is less stable.

Data for peroxidatic activity of monomeric cytoglobin Detailed descriptions of each data file can be found in the text file ‘Data description’ provided.

## Discussion

This study shows Cygb as having a high peroxidase-like activity in comparison to other pentacoordinate globins such as Mb. This is in contrast to previous reports that Cygb has a no appreciable peroxidase activity ^37^. This discrepancy could be explained by the different conformations of Cygb, with the current study using the monomeric protein with an internal disulfide bond between C38 and C83. Previous studies have not identified the conformation of the protein under investigation. We have previously shown that this disulfide bond is important for the biochemical properties of the protein with differences reported for ligand binding and redox chemistry ^20^. The nature of the Cygb conformation *in vivo* is unknown, however, at micromolar concentrations the protein is monomeric ^38^, which is consistent with the cellular concentrations.

Previous studies with Cygb have shown that mutation of the distal histidine affects ligand binding ^24^. The reaction of WT Cygb with peroxide showed the formation of the transient ferryl species ( Figure 5) and ferric-ferryl redox cycling ( Figure 4). This is in contrast to the H81V and H81Y mutants that appear not to exhibit ferryl or any significant guaiacol oxidation (<5% compared to WT). The lack of a distal histidine in Mb leads to a transient formation of peroxide-bound species (Compound 0) ^17^, which is not observed with our mutants of Cygb. However, the rapid degradation of haem as reported in the mutants by HPLC, stopped flow and EPR spectroscopy supports a conjecture that the proteins react fast with peroxide, but the ferryl formed in the reactions is extremely unstable as it is not observed in the millisecond timescale. This suggests that the lack of guaiacol oxidation in the H81V and H81Y proteins is due to the inability of the guaiacol to donate an electron to the ferryl before it decays. The instability of the ferryl could account for the extensive haem and protein damage and is in agreement with extensive protein radical formation on multiple free radical sites in the mutants as compared to the WT, resulting in unresolved singlet EPR signal ( Figure 7 and Figure 8).

The introduction of a tyrosine at the distal site increases resistance to peroxide-induced haem degradation as compared to valine, but degradation of the H81Y is still higher than that of the WT. This is likely due to the access to the haem being sterically hindered more by the larger cyclic amino acid. The polarity of the distal residue has also been suggested to affect the reaction with H _2_O _2_ - by providing a hydrogen bond to the ligand at the distal site ^17^. Both mutants show a partial low spin form ( Figure 3). This is not wholly unexpected for the H81Y mutant as the tyrosine could coordinate to the haem iron. However, the LS signal for this species is identical to that observed with H81V (but with different intensity), suggesting that another residue is coordinating to the iron. The EPR signal is similar to that observed in distal histidine mutants of Ngb ^39^. The identity of this residue is unknown, however; a nearby arginine (R84) is a position to potentially coordinate to the haem iron.

In summary, our study identifies the requirement of the distal histidine for the formation of a ferryl species in monomeric Cygb and prevention of rapid haem degradation by free radical activity. This is similar to what has been reported for distal histidine mutations (H64Q/H64V) in Mb ^17^. Monomeric Cygb with the internal disulfide bound has a weaker coordination of the distal histidine and is therefore more pentacoordinate-like. This is consistent with the EPR data ( Figure 3) that show partial penta-coordination which can also be increased through lipid binding ^19, 20^. This pentacoordinate-like nature of the Cygb allows for the reaction with peroxide, which is not observed with fully hexacoordinate Ngb. Although the ferryl Cygb is more unstable compared to this haem oxidation state in other pentacoordinate globins, the distal histidine introduces a resistance to free radical damage above that of other globins that show peroxidatic activity. These findings are consistent with a potential peroxidatic-like role of monomeric Cygb *in vivo*.

## Data availability

F1000Research: Dataset 1. Data for peroxidatic activity of monomeric cytoglobin, 10.5256/f1000research.5971.d45066 ^40^
