# Supplementary material for: Effect of the distal histidine on the peroxidatic activity of monomeric cytoglobin
Source: F1000Res. 2015 Apr 7;4:87. [Version 1] doi: 10.12688/f1000research.5971.1 (PMC4431388; doi:10.12688/f1000research.5971.1)
Supplement: Peer review history

# Peer review: Referee response for version 1

**DOI:** 10.5256/f1000research.6388.r8551

**Published:** 2015-05-01

**Authors:** Rakesh Pravinchandra Patel

**Affiliations:** 1 Department of Pathology and Center for Free Radical Biology and Lung Injury and Repair Center, University of Alabama at Birmingham, Birmingham, AL, USA

Cytoglobin in regulating redox reactions with H2O2. The study design and methods used are appropriate to address the proposed questions. The data nicely show that distal His residues of this protein play key roles in maintaining heme stability during reactions with H2O2.

1. Details of how many replicates and statistical analyses used to discern differences should be provided.
2. Is the heme loss observed after reactions with H2O2 and His mutants due to heme release and/ or heme modification that in turn would preclude extraction and measurement by HPLC.  The authors should provide some thoughts on how they think reactions with H2O2 (in the His mutants) is causing the heme to be released or degraded?
3. Are the effects of the distal His exclusive for reactions with H2O2? Could the authors speculate a little more on whether their results are more generalizable to other biologically relevant peroxides (LOOH, ONOO(H) etc.).

I have read this submission. I believe that I have an appropriate level of expertise to confirm that it is of an acceptable scientific standard.

# Peer review: Referee response for version 1

**DOI:** 10.5256/f1000research.6388.r8224

**Published:** 2015-04-28

**Authors:** Mark Hargrove

**Affiliations:** 1 Department of Biochemistry, Biophysics and Molecular Biology, Iowa State University, Ames, IA, USA

These authors have been publishing quality data with Cygb over the past few years, and the work presented here is a continuation of the same line of investigation.  The experiments conducted are fairly straight forward, and the immediate results are clear (with the possible exception of Figure 7, as discussed below), but the conclusions drawn from them are not as strong.  The goal of the work was to test the hypothesis that Cygb might serve as a peroxidase *in vivo*.  Since no one yet knows the actual function of Cygb *in vivo*, the present investigation must compare Cygb to something else.  Two point mutants of Cygb and myoglobin are used for comparison. 

Absorbance and EPR spectroscopy, along with reactions with H2O2, and a guaiacol-based peroxidase assay provide the bulk of the data to test this hypothesis.

**Interesting/positive points about this article:** Cygb and Mb react differently in the guaiacol assay (Cygb actually doesn’t react much at all). However, when an assay specific for formation of ferryl heme is used, both proteins react similarly (with respect to their bimolecular rate constants for the spectral change following mixing with H2O2). This means that Cygb can be oxidized by H2O2 just like Mb, but does not then oxidize a substrate such as guaiacol, as does Mb. 

**Remaining questions/criticisms of the article:** It is evident that interesting things happen when Cygb is mixed with H2O2, and that different things happen to the two point mutants. It is not at all clear what is learned from the EPR spectra. The conclusion is that the introduced Tyr might occlude R84 coordination, but this is very speculative and has little to do with the point of this article. 

Figure 7 is not an overwhelming endorsement of the stability of Cygb against hydrogen peroxide. The H2O2 dependence of Cygb modification is very strange, at first responding like Mb (for the first four points of the curve), then resisting the approach to 50% reduction of unmodified heme until the reported value of 2500 µM.  I think it is a bit misleading to judge these curves by the concentration of H2O2 needed to damage 50% of the heme, when the does-response is so complex. For example, if the parameter used to measure Mb versus Cygb were “concentration of H2O2 needed to damage 40% of the heme”, Mb and Cygb would be the same.

Why in the discussion is it said that Cygb has a higher peroxidase activity than Mb?  As measured by guaiacol, it does not, and as measured by the formation of the ferryl species, it is the same. Furthermore, the final conclusion for how these data test the primary hypothesis is that they are “consistent with a potential peroxidatic-like role of monomeric Cygb *in vivo”. *While these results are interesting, I am not sure how they could fail in support of this hypothesis, short of Cygb not reacting at all with H2O2.  Based on the data reported here, these results also support a role for Mb having a peroxidatic-like activity *in vivo*.

I have read this submission. I believe that I have an appropriate level of expertise to confirm that it is of an acceptable scientific standard.
